# Advanced Airway Devices and End-Tidal Capnography Trends in Cardiac Arrest: A Secondary Analysis of a Randomized Clinical Trial

**DOI:** 10.1001/jamanetworkopen.2025.31511

**Published:** 2025-09-15

**Authors:** Michelle M. J. Nassal, Andoni Elola, Elisabete Aramendi, Christopher B. Gage, Jonathan R. Powell, Xabier Jaureguibeitia, Ahamed H. Idris, Mohamud R. Daya, Tom P. Aufderheide, Jestin Carlson, Shannon W. Stephens, Graham Nichol, Robert H. Schmicker, Ashish R. Panchal, Henry E. Wang

**Affiliations:** 1 Department of Emergency Medicine, The Ohio State University, Columbus; 2 Department of Electronic Technology, BioRes Group, University of the Basque Country, Universidad del País Vasco/Euskal Herriko Unibertsitatea (UPV/EHU), Bilbao, Spain; 3 Department of Communication Engineering, BioRes Group, University of the Basque Country, UPV/EHU, Bilbao, Spain; 4 National Registry of Emergency Medical Technicians, Columbus, Ohio; 5 ImageTrend, LLC, Eagan, Minnesota; 6 Department of Emergency Medicine, University of Texas Southwestern Medical Center, Dallas; 7 Department of Computer Science and Engineering, Emergency Medicine, The Ohio State University, Columbus; 8 Department of Emergency Medicine, Oregon Health & Science University, Portland; 9 Department of Emergency Medicine, Medical College of Wisconsin, Milwaukee; 10 Department of Emergency Medicine, University of Pittsburgh, Pittsburgh, Pennsylvania; 11 Harborview Center for Prehospital Emergency Care, University of Washington, Seattle; 12 University of Washington Clinical Trial Center, Seattle

## Abstract

**Importance:**

While variable advanced airway devices are used in out-of-hospital cardiac arrest (OHCA) resuscitation, potential differences in ventilation metrics are not well known.

**Objective:**

To characterize differences in end-tidal carbon dioxide (EtCO_2_) capnography trajectories between laryngeal tube (LT) and endotracheal intubation (ETI) during OHCA.

**Design, Setting, and Participants:**

This secondary analysis of EtCO_2_ capnography waveforms from the Pragmatic Airway Resuscitation Trial (PART), a multicenter cluster-crossover randomized clinical trial, was performed November 1, 2023, through July 8, 2025. Participants in this analysis were adults (aged ≥18 years) with nontraumatic OHCA from 27 emergency medical services (EMS) agencies, which were placed into 13 clusters. Participants were assigned to LT or ETI airway management. All cases with 50% or greater interpretable EtCO_2_ signal were included.

**Interventions:**

LT vs ETI airway management.

**Main Outcomes and Measures:**

Mean maximal EtCO_2_ values within 1-minute epochs and trends over resuscitation after LT or ETI. Associations with OHCA outcomes were also evaluated, including sustained return of spontaneous circulation (ROSC) and 72-hour survival. Using the Mann-Whitney test, EtCO_2_ of LT and ETI cases were compared at 20-, 10-, and 1-minute resuscitation. EtCO_2_ trends over time were evaluated using Cochran-Armitage test of trend. Covariates in multivariable logistic regression models for outcomes were adjusted, including interaction between advanced airway device and EtCO_2_ trends.

**Results:**

Of the 3004 cases in PART, 1113 (818 in LT and 295 in ETI groups) had available EtCO_2_ data and were included in the secondary analysis. Patients were mostly male (694 [62.4%]), had a median (IQR) age of 64 (52-75) years, and had a nonshockable (941 [84.6%]), nonpublic (999 [89.8%]) OHCA. ROSC occurred in 144 patients (17.6%) receiving LT and 54 (18.3%) receiving ETI. EtCO_2_ values did not differ between LT and ETI groups (20-minute resuscitation: 33.9 vs 29.4 mm Hg, *P* = .07; 10-minute resuscitation: 30.9 vs 28.5 mm Hg, *P* = .89; 1-minute resuscitation: 32.2 vs 28.3 mm Hg, *P* = .28). In ROSC compared with non-ROSC cases, patients in LT (27.9 to 52.3 mm Hg vs 32.6 to 23.5 mm Hg; *P* < .001) and ETI (38.2 to 46.7 mm Hg vs 27.7 to 20.0 mm Hg; *P* < .001) groups exhibited increasing EtCO_2_ during resuscitation. EtCO_2_ trend and advanced airway interaction was associated with ROSC (odds ratio [OR], 1.75; 95% CI, 1.25-2.45) but not survival (OR, 1.21; 95% CI, 0.90-1.61). Therefore, we performed an advanced airway stratified analysis for ROSC (ETI: OR, 2.34 [95% CI, 1.67-3.26]; LT: OR, 1.33 [95% CI, 1.20-1.47]).

**Conclusions and Relevance:**

In this secondary analysis of PART, EtCO_2_ values did not differ between LT and ETI. However, due to significant interaction between advanced airway device and EtCO_2_ trends, individual interpretation of these parameters may not be accurate.

## Key Points

**Question:**

Does the end-tidal carbon dioxide (EtCO_2_) capnography trend differ by type of advanced airway device placed and does this difference affect outcomes of out-of-hospital cardiac arrest?

**Findings:**

In this secondary analysis involving 1113 participants in the Pragmatic Airway Resuscitation Trial, EtCO_2_ values over resuscitation duration were not different by advanced airway device. The type of advanced airway device interacted with the EtCO_2_ capnography trend; however, both devices had similar ability to estimate return-of-spontaneous circulation in out-of-hospital cardiac arrest.

**Meaning:**

Findings of this study suggest that EtCO_2_ capnography trends during resuscitation are directly associated with outcomes in both laryngeal tube and endotracheal intubations.

## Teaser

This secondary analysis of the Pragmatic Airway Resuscitation Trial examines the association between the type of advanced airway device used, end-tidal carbon dioxide trajectories during resuscitation, and outcomes in adults with nontraumatic out-of-hospital cardiac arrest.

## Introduction

Out-of-hospital cardiac arrest (OHCA) remains a leading cause of morbidity and mortality globally.^1^ Current resuscitation guidelines recommend advanced airway placement, such as endotracheal intubation (ETI) and supraglottic airway (SGA) insertion, during OHCA resuscitation.^2^ However, previous studies suggest potential outcome differences between SGA and ETI.^3,4,5^ Observational studies found ETI to be associated with improved OHCA outcomes.^5^ The Pragmatic Airway Resuscitation Trial (PART) found that compared with ETI, laryngeal tube SGA was associated with improved 72-hour survival.^3^ In contrast, the AIRWAYS-2 trial found no association between ETI and i-gel SGA and 30-day functional recovery.^4^ The purpose of advanced airway devices is to facilitate oxygen delivery and ventilation. Ventilation effectiveness may explain these variable findings.

SGA and ETI have key differences that could alter the effectiveness of delivered ventilation. They are structurally different; offer varying passageways to the trachea; and provide variable oxygenation, ventilation, and pressure control.^6,7^ SGA includes devices with inflatable cuffs (such as the laryngeal tube (LT) or laryngeal mask airway) and noninflatable cuffs (such as the i-gel) that fill the perilaryngeal and hypopharyngeal areas for ventilation.^8^ In contrast, ETI is positioned directly between the vocal cords and has an inflatable cuff to facilitate a seal for ventilation. End-tidal carbon dioxide (EtCO_2_) capnography is used with both devices to confirm advanced airway placement. Furthermore, EtCO_2_ may indicate ventilation quality and impending return of spontaneous circulation.^2^ Previous studies have shown that rapid increases in EtCO_2_ are associated with return of spontaneous circulation (ROSC) and 72-hour survival.^9,10^ The distinctions in EtCO_2_ measurements between advanced airway devices have not been well characterized.

We sought to characterize differences in EtCO_2_ trajectories between LT and ETI during OHCA in PART. We also sought to determine whether the type of advanced airway device altered the association between EtCO_2_ trends and OHCA outcomes.

## Methods

### Design, Setting, Participants

This secondary analysis of EtCO_2_ capnography waveforms from PART^3^ was performed from November 1, 2023, through July 8, 2025. The trial was registered through ClinicalTrials.gov (NCT02419573). The trial itself, conducted from December 1, 2015, to November 4, 2017, used a cluster-crossover design and enrolled participants from 27 emergency medical services (EMS) agencies in 5 communities of the Resuscitation Outcomes Consortium in North America. The institutional review boards of the participating EMS agencies approved the trial and waived the informed consent requirement under federal rules for conduct of emergency research under Exception from Informed Consent.^3^ The trial protocol is available in Supplement 1. For this post hoc analysis, we included only PART participants with successful advanced airway placement and available continuous capnography data. The Ohio State University Institutional Review Board approved this secondary analysis. We followed the Consolidated Standards of Reporting Trials (CONSORT) reporting guideline.^11^

PART assigned adults (aged ≥18 years) with nontraumatic OHCA to strategies of LT or ETI airway management. The trial excluded patients who were younger than 18 years, were pregnant, were in prison, or had traumatic OHCA. EMS agencies were placed into 13 clusters. Computer randomization occurred in blocks of 2 within each cluster and a priori determined crossover intervals ranging from 3 to 5 months that was implemented by the lead statistician in PART.^3^ The trial did not limit the number of advanced airway attempts or rescue devices used after a failed advanced airway placement. Demographic data, including race and ethnicity, were reported through electronic health records of the EMS agencies. Race and ethnicity (categorized as Black or African American, White, and other [including American Indian or Alaska Native, Asian, Hispanic, Native Hawaiian or Pacific Islander, and unknown or not noted]) were included in the analysis because race is a known factor in OHCA.^12^ The trial enrolled a total of 3004 patients.

### Measures

The primary measure of this analysis was EtCO_2_. As part of their standard care, EMS agencies collected continuous EtCO_2_ capnography waveforms using portable cardiac defibrillator monitors (LifePak 15 series, Physio-Control Inc; X-series, Zoll Medical Corp; and MRx series, Philips Healthcare). We annotated the maximal EtCO_2_ value for each ventilation using previously validated automated signal processing.^13^ Import and analysis of capnography waveforms were accomplished using MATLAB (Mathworks Inc), and a custom graphical user interface was used for case revision.^14,15^ We collected mean EtCO_2_ values in 1-minute epochs. We included all cases with greater than 50% interpretable EtCO_2_ signal in at least 1 of the epochs.

### Outcomes

Our primary outcome was EtCO_2_ values within 1-minute epochs and trends over resuscitation duration. We also determined associations with OHCA outcomes, including sustained ROSC and 72-hour survival. ROSC was determined by clinical evaluation in PART.^3^ Research coordinators who were ascertaining clinical outcomes were not blinded to the study interventions in PART.

### Statistical Analysis

To evaluate potential differences in EtCO_2_, we stratified our analysis based on the last successful advanced airway placed: LT or ETI. We developed histogram plots to show the distribution of EtCO_2_ values in all cases. We compared discrete time point measurements of EtCO_2_ between LT and ETI using the Mann-Whitney test. We also developed box plots depicting the median (IQR) values of EtCO_2_ at each minute epoch over 20 minutes of resuscitation prior to the end of the event. We determined the association between temporal trends in EtCO_2_ using Cochran-Armitage test of trend. We calculated the slope of EtCO_2_ by change in EtCO_2_ over sequential minutes in the resuscitation period (mm Hg/min) as previously described.^9^ We then performed a multivariable logistic regression model for outcomes that was adjusted for the slope of EtCO_2_, interaction between EtCO_2_ trends and advanced airway device, age, sex, public location, witnessed cardiac arrest (bystander-witnessed, EMS-witnessed, or unwitnessed), bystander cardiopulmonary resuscitation (CPR; yes or no), and initial electrocardiogram rhythm (shockable vs nonshockable). We evaluated the interaction between EtCO_2_ trends and advanced airway device because we hypothesized potential differences in ventilation from advanced airway devices. For a sensitivity analysis, we repeated the primary analysis using generalized estimating equations to account for the randomized cluster trial design. We assessed goodness-of-fit testing using Hosmer-Lemeshow statistics.

Statistical significance was considered with 2-sided *P* < .05. Analysis was conducted using Stata, version 16.0 (StataCorp LLC).

## Results

**Figure 1.**
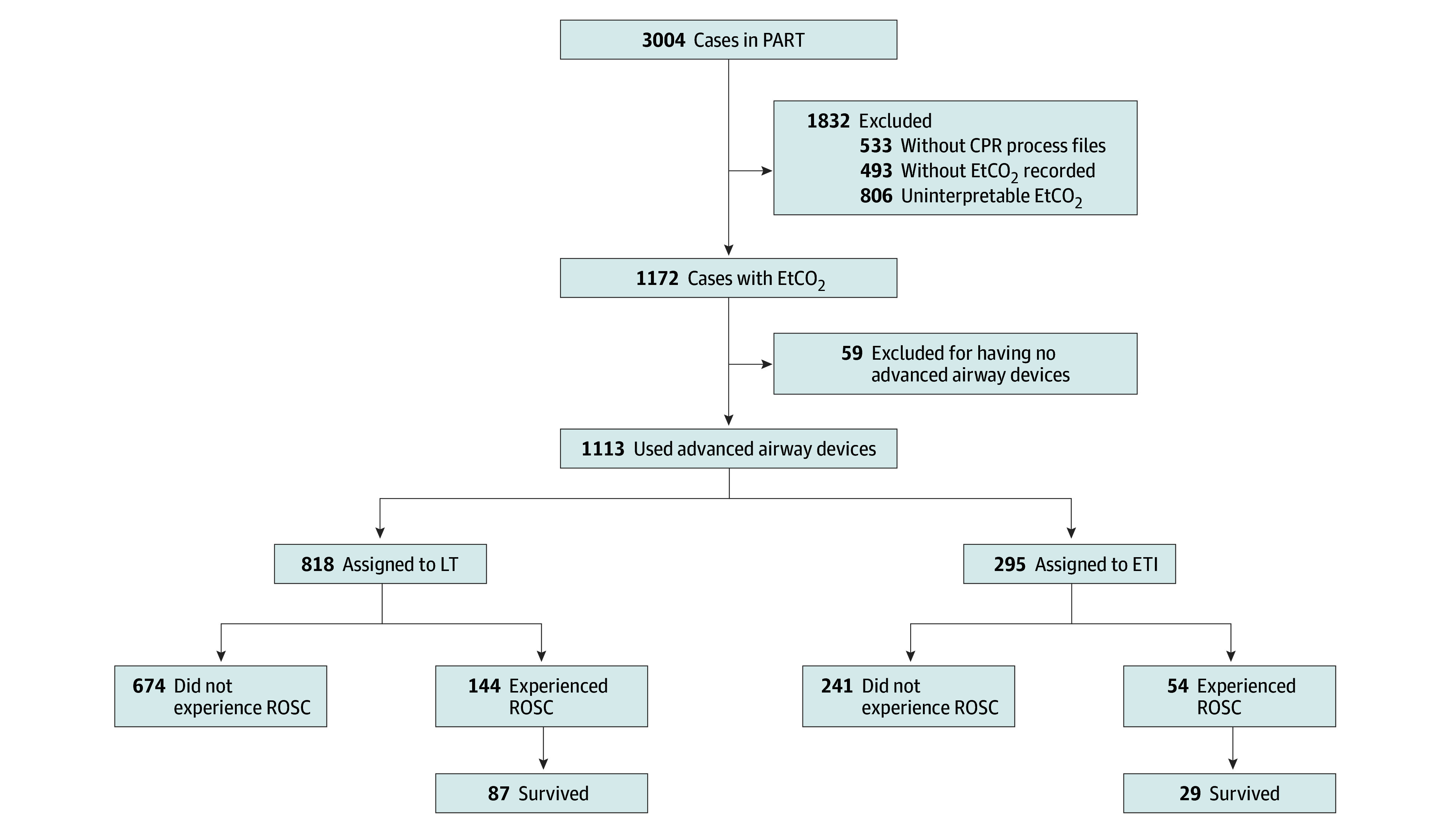
Cases Included in the Analysis Analysis limited to 1172 patients in the Pragmatic Airway Resuscitation Trial (PART) with end-tidal carbon dioxide (EtCO_2_) capnography recordings. CPR indicates cardiopulmonary resuscitation; ETI, endotracheal intubation; LT, laryngeal tube; and ROSC, return of spontaneous circulation.

Of the 3004 cases included in PART, 1113 (37.1%) had available EtCO_2_ recorded using an advanced airway device (Figure 1) and were included in the present analysis. These patients included 694 males (62.4%) and 419 females (37.6%) with a median (IQR) age of 64 (52-75) years, of whom 285 were identified as Black or African American (25.6%), 592 as White (53.2%), and 236 as other (21.2%) race and ethnicity (Table 1). Among them, 941 had a nonshockable (84.6%) and 999 had a nonpublic OHCA (89.8%). The majority of advanced airway devices were LT (818 [73.5%]), with ROSC occurring in 144 patients (17.6%) receiving LT and 54 patients (18.3%) receiving ETI.

**Table 1.** Demographic Characteristics of Included Cases

Characteristic	PART cohort, No. (%)			Present cohort, No. (%)		
	Total (n = 3004)	LT group (n = 1351)	ETI group (n = 542)	Total (n = 1113)	LT group (n = 818)	ETI group (n = 295)
Age, median (IQR), y	64 (53-76)	63 (51-74)	66 (56-77)	64 (52-75)	63 (51-74)	66 (55-79)
Sex						
Female	1175 (39.1)	500 (37.0)	228 (42.0)	419 (37.6)	294 (35.9)	125 (42.4)
Male	1829 (60.9)	851 (63.0)	314 (57.9)	694 (62.4)	524 (64.1)	170 (57.6)
Race and ethnicity^a^						
Black or African American	863 (28.7)	369 (27.3)	186 (34.2)	285 (25.6)	203 (24.8)	82 (27.8)
White	1542 (51.33)	703 (52.0)	256 (47.2)	592 (53.2)	437 (53.4)	155 (52.5)
Other^b^	599 (19.9)	279 (21.0)	100 (18.5)	236 (21.2)	178 (21.8)	58 (19.7)
Location of cardiac arrest						
Public	352 (11.7)	139 (10.3)	63 (11.6)	113 (10.2)	77 (9.4)	36 (12.2)
Nonpublic	2645 (88.1)	1208 (89.4)	478 (88.2)	999 (89.8)	740 (90.5)	259 (87.8)
Missing data	7 (0.2)	4 (0.3)	1 (0.2)	1 (0.1)	1 (0.1)	0
Witnessed status						
Unwitnessed	1357 (45.1)	633 (46.9)	248 (45.7)	579 (52.0)	425 (52.0)	154 (52.2)
Bystander-witnessed	1040 (34.6)	460 (34.1)	194 (35.8)	327 (29.4)	236 (28.9)	91 (30.9)
EMS-witnessed	359 (11.9)	131 (9.7)	28 (5.2)	102 (9.2)	86 (10.5)	16 (5.4)
Missing data	248 (8.3)	127 (9.4)	72 (13.8)	105 (9.4)	71 (8.7)	34 (11.5)
Initial rhythm						
Shockable	571 (19.0)	216 (16.0)	93 (17.2)	172 (15.5)	126 (15.4)	46 (15.6)
Nonshockable	2357 (78.5)	1135 (84.1)	449 (82.8)	941 (84.6)	692 (84.6)	249 (84.4)
Bystander CPR	1407 (46.8)	629 (45.6)	257 (47.4)	556 (50.0)	400 (48.9)	157 (53.2)
Bystander AED	295 (9.8)	137 (10.1)	61 (11.3)	121 (11.0)	84 (10.3)	37 (12.5)
ROSC	785 (26.1)	316 (23.4)	139 (25.7)	198 (17.8)	144 (17.6)	54 (18.3)
Survival	505 (16.8)	191 (14.1)	74 (13.6)	116 (10.4)	87 (10.6)	29 (9.8)

Abbreviations: AED, automatic external defibrillator; CPR, cardiopulmonary resuscitation; EMS, emergency medical services; ETI, endotracheal intubation; LT, laryngeal tube; PART, Pragmatic Airway Resuscitation Trial; ROSC, return of spontaneous circulation.

^a^ Race and ethnicity reported in electronic health records and emergency medical services agencies.

^b^ Other includes American Indian or Alaska Native, Asian, Hispanic, Native Hawaiian or Pacific Islander, and unknown or not noted. Categories were combined following the convention in previous studies.

**Figure 2.**
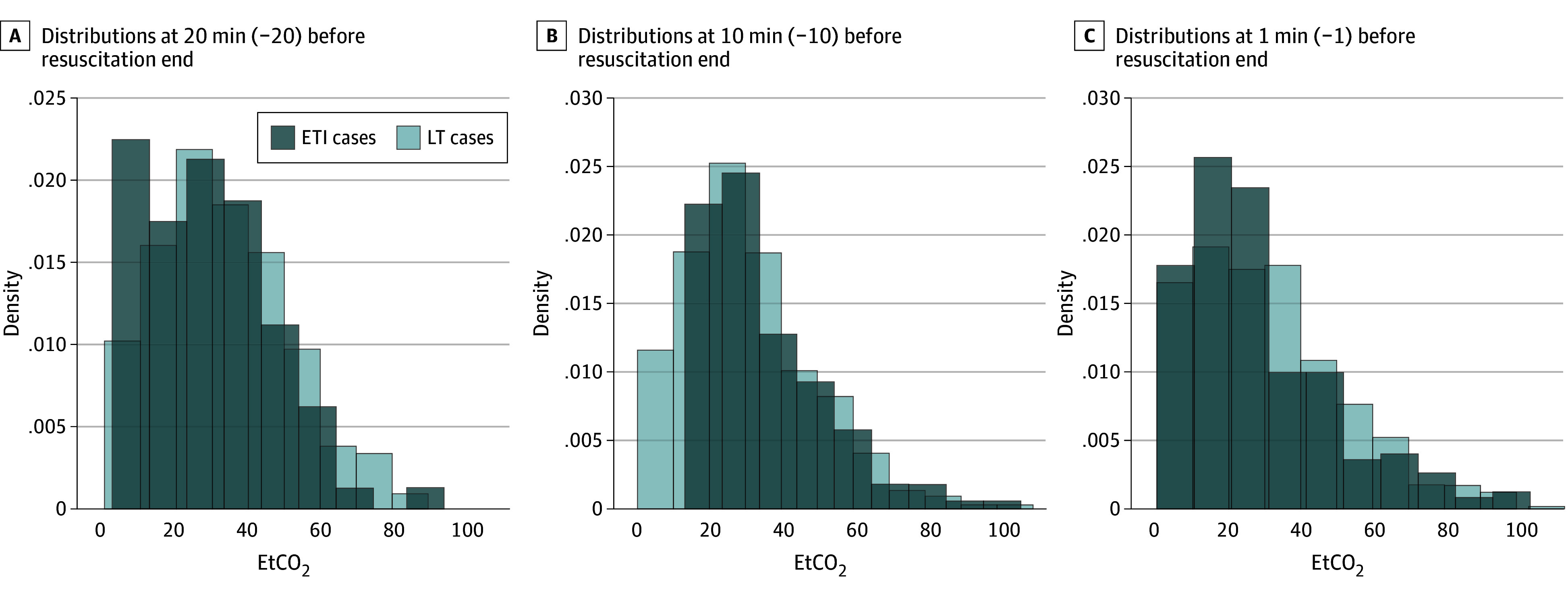
End-Tidal Carbon Dioxide (EtCO_2_) Capnography vs Time ETI indicates endotracheal intubation; LT, laryngeal tube.

**Figure 3.**
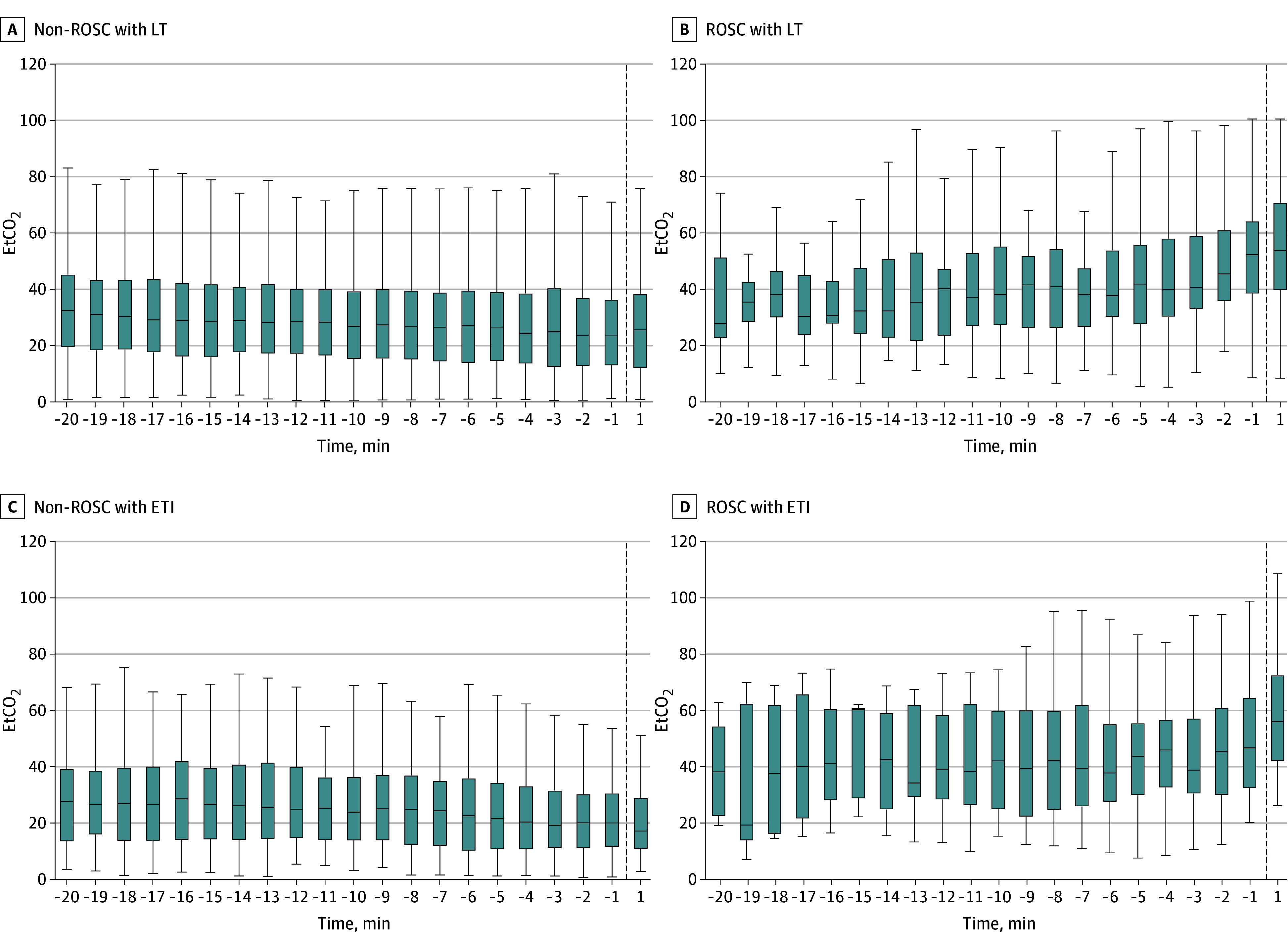
End-Tidal Carbon Dioxide (EtCO_2_) Capnography vs Time, Stratified by Advanced Airway Technique Box plots describe EtCO_2_ capnography values in 1-minute epochs. Vertical dashed line indicates end of resuscitation. ETI indicates endotracheal intubation; LT, laryngeal tube; and ROSC, return of spontaneous circulation.

Median (IQR) capnography recorded was similar between LT (10 [5-17] minutes) and ETI (11 [6-19] minutes) cases. Before achieving ROSC, EtCO_2_ values did not differ between LT and ETI groups (20-minute resuscitation: 33.9 vs 29.4 mm Hg, *P* = .07; 10-minute resuscitation: 30.9 vs 28.5 mm Hg, *P* = .89; 1-minute resuscitation: 32.2 vs 28.3 mm Hg, *P* = .28) (Figure 2). In cases achieving ROSC compared with non-ROSC, patients in both LT (27.9 to 52.3 mm Hg vs 32.6 to 23.5 mm Hg; *P* < .001) and ETI (38.2 to 46.7 mm Hg vs 27.7 to 20.0 mm Hg; *P* < .001) groups exhibited increasing EtCO_2_ during resuscitation (Figure 3).

In the multivariable model estimating ROSC, the multiplicative interaction between EtCO_2_ slope and advanced airway device was significant (odds ratio [OR], 1.75; 95% CI, 1.25-2.45; *P* < .001) but was not significant for survival (OR, 1.21; 95% CI, 0.90-1.61) (Table 2). Calibration for models was acceptable (ROSC, 0.75; survival, 0.78). Generalized estimating equation modeling to account for the trial randomization clusters produced similar results. For patients with final airway ETI compared with LT the odds of ROSC were greater (OR, 2.34 [95% CI, 1.67-3.26] vs 1.33 [95% CI, 1.20-1.47]) (eAppendix in Supplement 2). Calibration for models was similarly acceptable (ETI, 0.79; LT, 0.75).

**Table 2.** Associations Between End-Tidal Carbon Dioxide Temporal Slope, Advanced Airway Device, and Cardiac Arrest Outcomes

Variable	ROSC, OR (95% CI)	*P* value	Survival, OR (95% CI)	*P* value
EtCO_2_ slope^a^	1.34 (1.21-1.48)	<.001	1.22 (1.09-1.36)	<.001
Advanced airway device				
LT	1 [Reference]		1 [Reference]	
ETI	0.94 (0.64-1.52)	.81	0.78 (0.43-1.44)	.43
EtCO_2_ slope × advanced airway device	1.75 (1.25-2.45)	.001	1.21 (0.90-1.61)	.20
Age	0.99 (0.98-1.00)	.11	0.98 (0.97-1.00)	.01
Male sex	0.87 (0.58-1.29)	.48	0.84 (0.51-1.39)	.50
Public cardiac arrest location	1.86 (1.08-3.21)	.03	2.02 (1.11-3.68)	.02
Shockable rhythm	2.88 (1.86-4.46)	<.001	4.57 (2.76-7.58)	<.001
Bystander CPR	0.98 (0.68-1.45)	.97	0.95 (0.58-1.53)	.82
Bystander-witnessed	1.70 (1.30-2.23)	<.001	1.84 (1.32-2.58)	<.001

Abbreviations: CPR, cardiopulmonary resuscitation; EtCO_2_, end-tidal carbon dioxide; ETI, endotracheal intubation; LT, laryngeal tube; OR, odds ratio; ROSC, return of spontaneous circulation.

^a^ Change in EtCO_2_ over resuscitation calculated as the slope of EtCO_2_ (mm Hg/min).^9^

In the primary analysis, we classified participants according to advanced airway device received. In the sensitivity analysis, we repeated the main analysis by intention-to-treat groups. We found a persistent association between EtCO_2_ slope and ROSC in both LT (OR, 1.45; 95% CI, 1.27-1.65) and ETI groups(OR, 1.41; 95% CI, 1.22-1.64). Full regression model outputs are available in the eAppendix in Supplement 2.

## Discussion

EtCO_2_ is a potentially important biosignal in OHCA. In this study, we examined possible differences in EtCO_2_ trends between advanced airway devices during OHCA resuscitation. We observed that discrete EtCO_2_ values did not differ by advanced airway device. We also observed that EtCO_2_ trends over resuscitation time appeared similar between LT and ETI. For the outcome of ROSC, we observed an interaction between advanced airway device and EtCO_2_ slope over resuscitation duration. Our results suggest that there may be some unmeasured differences in the effectiveness of ventilation between advanced airway devices.

The most important finding of this analysis is that there is an interaction between the type of advanced airway device used, EtCO_2_ trends, and outcomes. This observation suggests there may be differences in capnography trajectories between ETI and LT. While both advanced airway devices showed direct associations between slope of EtCO_2_ and ROSC, they may have differentially affected CPR circulatory dynamics. For example, increased intrathoracic pressure may increase or decrease coronary perfusion pressure during CPR. These hypothesis-generating observations suggest there could be additional unidentified effects of advanced airway device on EtCO_2_ trends and OHCA outcomes. Because we used EtCO_2_ trends in resuscitation decisions, these data emphasize that increasing trajectories in capnography support continued efforts as this trend is likely to be associated with ROSC from any advanced airway device.

An important consideration in this analysis is that LT cases included failed first–ETI attempts. Thus, interactions between advanced airway device and slope of EtCO_2_ with ROSC could have been altered by the duration of resuscitation efforts. When we repeated the analysis by intention-to-treat groups, the association between EtCO_2_ slope and ROSC no longer differed between advanced airway device groups. Thus, the ETI association in the as-treated, primary analysis may reflect cases with first-pass intubation success. Furthermore, the interaction between advanced airway device and slope of EtCO_2_ significantly affected associations with ROSC but not with 72-hour survival; this primary analysis lacked precision for survival because of the small sample size (n = 116: 87 for LT, 29 for ETI). Additional investigation into potential variability in advanced airway device and changes in EtCO_2_ values during the course of resuscitation is warranted.

Capnography is one potential marker for ventilation quality as it is also useful in documenting ventilation counts.^16^ This secondary analysis and other studies determined that improved survival was not likely associated with varying advance airway ventilation rates or EtCO_2_ trends.^9,17^ Prior studies also found that the rates of first-pass success with LT did not explain improved OHCA outcomes.^18,19^ The underlying reasons for improved outcomes with LT use, as observed in PART, remains unknown. We cannot evaluate other markers of ventilation effectiveness such as tidal volume and pressure, which may vary by advanced airway device.^7^ Other potential contributions may include timing of advanced airway placement,^19^ variable intrathoracic pressure changes, or a combination of these factors.

Capnography use during resuscitation remains vital given that few other real-time biomarkers exist to guide prehospital resuscitation. The feasibility of capnography monitoring paired with the established association of EtCO_2_ with OHCA outcomes has supported continued capnography during resuscitation.^20,21^ These data offer clinical information that ETI and LT may provide similar ventilation characteristics when clinicians consider advanced airway options during resuscitation.

### Limitations

There are considerable limitations when considering these findings. Although EtCO_2_ serves as a surrogate for ventilation effectiveness, its use as a biomarker for ventilation-perfusion exchange is unclear when circulation status varies.^22,23^ Capnography is affected by resuscitation quality, specifically chest compression quality and ventilation rates.^24,25,26,27^ Medications are also known to affect capnography.^28,29^ Lastly, only 39.0% (1172 of 3004) of PART participants had available capnography for evaluation. Comparing OHCA characteristics to those in PART, this analysis appeared to have slightly higher number of unwitnessed nonshockable cardiac arrests, which could contribute to worse overall outcomes than in PART. However, other patient demographic and OHCA characteristics were comparable (Table 1). We also did not assess the number of advanced airway attempts in this analysis, which may have affected capnography, although we found similar capnography data availability in LT and ETI (10-11 minutes) cases.

## Conclusions

In this secondary analysis of a randomized clinical trial, discrete EtCO2 values did not differ by advanced airway device during OHCA resuscitation. These results support that ventilation may be similar between advanced airway types. However, the associations between EtCO2 trajectory and outcomes differed between advanced airway devices. Therefore, individual interpretation of these parameters may not be accurate, highlighting the need for additional studies.

## Notes

Supplement 1.
Clinical Trial Protocol

Supplement 2. **eAppendix.** Stratified Analysis Evaluating Slope of EtCO2 by Advanced Airway Device

Supplement 3.
Data Sharing Statement
