# Supplementary material for: Advanced Airway Devices and End-Tidal Capnography Trends in Cardiac Arrest: A Secondary Analysis of a Randomized Clinical Trial
Source: JAMA Netw Open. 2025 Sep 15;8(9):e2531511. doi: 10.1001/jamanetworkopen.2025.31511 (PMC12439061; doi:10.1001/jamanetworkopen.2025.31511)
Supplement: Supplement 2. — eAppendix. Stratified Analysis Evaluating Slope of EtCO2 by Advanced Airway Device [file jamanetwopen-e2531511-s002.pdf]

Supplemental Online Content

Nassal MMJ, Elola A, Aramendi E, et al. Advanced airway devices and end-tidal capnography trends in cardiac arrest: a secondary analysis of a randomized clinical trial. *JAMA Netw Open*. 2025;8(9):e2531511. doi:10.1001/jamanetworkopen.2025.31511

eAppendix. Stratified Analysis Evaluating Slope of $EtCO_2$ by Advanced Airway Device

This supplemental material has been provided by the authors to give readers additional information about their work.

© 2025 Nassal MMJ et al. *JAMA Network Open*

eAppendix. Stratified Analysis Evaluating Slope of $EtCO_2$ by Advanced Airway Device

Left Columns show As-Treated analysis with final successful advanced airway placement. Right Columns show Intention-to-treat analysis. . Odds ratios (OR) with 95% CI shown. Significant levels: * p<0.05, *** p<0.001

**Variable**	**Stratified Analysis: Mixed Model for ROSC; As-Treated**		**Stratified Analysis: Mixed Model for ROSC; Intention to Treat**	
	LT (n=738)	ETI (n=254)	LT (n=532)	ETI (n=500)
Slope of $EtCO_2$	1.33 (1.20, 1.47) ***	2.34 (1.67, 3.26) ***	1.45 (1.27, 1.65) ***	1.41 (1.22, 1.64) ***
Age	0.99 (0.98, 1.00)	0.98 (0.97, 1.00) *	0.98 (0.97, 0.99)***	1.01 (0.99, 1.02) *
Male Sex	0.85 (0.54, 1.34)	0.84 (0.51, 1.39)	1.04 (0.62, 1.74)	0.56 (0.31, 1.02)
Public Location	1.43 (0.76, 2.69)	2.02 (1.11, 3.68) *	1.23 (0.61, 2.5)	3.72 (1.67, 8.30) ***
Shockable Rhythm	3.27 (2.01, 5.30) ***	4.57 (2.76, 7.58)***	3.22 (1.87, 5.54) ***	2.59 (1.29, 5.22)***
Bystander CPR	0.87 (0.57, 1.33)	0.95 (0.58, 1.53)	0.87 (0.54, 1.40)	1.53 (0.83, 2.83)
Bystander Witnessed	1.68 (1.26, 2.26) ***	1.84 (1.32, 2.58) ***	1.44 (1.03, 2.03) *	2.11 (1.22, 1.64) ***

© 2025 Nassal MMJ et al. *JAMA Network Open*
