# Supplementary material for: Advanced Airway Devices and End-Tidal Capnography Trends in Cardiac Arrest: A Secondary Analysis of a Randomized Clinical Trial
Source: JAMA Netw Open. 2025 Sep 15;8(9):e2531511. doi: 10.1001/jamanetworkopen.2025.31511 (PMC12439061; doi:10.1001/jamanetworkopen.2025.31511)
Supplement: Supplement 3. — Data Sharing Statement [file jamanetwopen-e2531511-s003.pdf]

Data Sharing Statement

Nassal. Advanced Airway Devices and End-Tidal Capnography Trends in Cardiac Arrest.
JAMA Netw Open. Published September 15, 2025. doi:10.1001/jamanetworkopen.2025.31511

Data
Additional Information: Trial Registration ClinicalTrials.gov Identifier: NCT02419573
Data available: Yes
Data types: Deidentified participant data
How to access data: BioLINCC
When available: beginning date: 06-26-2019

Supporting Documents
Document types: None

Additional Information
Who can access the data: anyone requesting the data
Types of analyses: for any purpose
Mechanisms of data availability: request through BioLINCC
